# Analysis of vaginal and endometrial microbiota communities in infertile women with a history of repeated implantation failure

**DOI:** 10.1002/rmb2.12389

**Published:** 2021-05-31

**Authors:** Takuhiko Ichiyama, Keiji Kuroda, Yoko Nagai, Daichi Urushiyama, Motoharu Ohno, Takashi Yamaguchi, Motoi Nagayoshi, Yoshiyuki Sakuraba, Fumio Yamasaki, Kenichiro Hata, Shingo Miyamoto, Atsuo Itakura, Satoru Takeda, Atsushi Tanaka

**Affiliations:** ^1^ Saint Mother Obstetrics and Gynecology Clinic and Institute for Assisted Reproductive Technologies Fukuoka Japan; ^2^ Department of Obstetrics and Gynecology Juntendo University School of Medicine Tokyo Japan; ^3^ Center for Reproductive Medicine and Implantation Research Sugiyama Clinic Shinjuku Tokyo Japan; ^4^ Varinos Inc. Tokyo Japan; ^5^ Department of Maternal‐Fetal Biology National Research Institute for Child Health and Development Tokyo Japan; ^6^ Department of Obstetrics and Gynecology Faculty of Medicine Fukuoka University Fukuoka Japan; ^7^ Division of Pathology Japan Community Health Care Organization Saga Central Hospital Saga Japan

**Keywords:** 16S rRNA, bacterial vaginosis, dysbiosis, microbiome, microbiota, repeated implantation failure

## Abstract

**Purpose:**

To identify specific bacterial communities in vaginal and endometrial microbiotas as biomarkers of implantation failure by comprehensively analyzing their microbiotas using next‐generation sequencing.

**Methods:**

We investigated α‐ and β‐diversities of vaginal and endometrial microbiotas using 16S rRNA gene sequencing and compared their profiles between 145 women with repeated implantation failure (RIF) and 21 controls who lacked the factors responsible for implantation failure with a high probability of being healthy and fertile to identify specific bacteria that induce implantation failure.

**Results:**

The endometrial microbiotas had higher α‐diversities than did the vaginal microbiotas (*P* < .001). The microbiota profiles showed that vaginal and endometrial samples in RIF patients had significantly higher levels of 5 and 14 bacterial genera, respectively, than those in controls. Vaginal *Lactobacillus* rates in RIF patients were significantly lower at 76.4 ± 38.9% compared with those of the controls at 91.8 ± 22.7% (*P* = .018), but endometrial *Lactobacillus* rates did not significantly differ between the RIF patients and controls (56.2 ± 36.4% and 58.8 ± 37.0%, respectively, *P* = .79).

**Conclusions:**

Impaired microbiota communities containing specific bacteria in both the endometrium and vagina were associated with implantation failure. The vaginal *Lactobacillus* rates, but not the endometrial, may be a biomarker for RIF.

## Graphical abstract

Vaginal and endometrial microbiotas might be independent. Endometrial microbiota has a richer alpha diversity than vaginal microbiota, and dysbiosis of vaginal or endometrial microbiota can cause implantation failure. Unlike previous reports, the *Lactobacillus* rate in the vagina, but not the endometrium, may be a biomarker for repeated implantation failure.

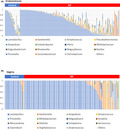

## 1 INTRODUCTION

In vitro fertilization (IVF) technology and quality have rapidly advanced. Recent reports of preimplantation genetic testing for aneuploidy reported >60% clinical pregnancy rates after embryo transfer (ET) cycles.^1^, ^2^ However, pregnancy requires competent embryos and a receptive endometrium; therefore, repeated implantation failure (RIF) with euploid embryos is difficult to treat.^3^

Reproductive‐related microbiota communities in women can affect reproductive and obstetric outcomes.^4^ Bacterial vaginosis (BV) is associated with obstetric complications, including pregnancy loss and preterm birth.^5^, ^6^, ^7^, ^8^, ^9^, ^10^ Analyzing microbiota profiles of the amniotic fluid may help predict perinatal outcomes.^11^ Moreno et al^12^ revealed vaginal and endometrial bacterial communities from vaginal aspirates and endometrial fluid from fertile and infertile women who underwent IVF. Data from the endometrial samples showed that bacterial communities from women experiencing implantation failure or pregnancy loss after ET contained more *Gardnerella* and *Streptococcus* and fewer *Lactobacillus* than did those from women who had successful livebirths. Furthermore, infertile patients with >90% *Lactobacillus* in their endometrial microbiota (EM) had significantly good pregnancy prognoses after IVF than did those with <90%. Therefore, endometrial microbiome analyses are used to determine individual EM profiles in infertile women.^13^, ^14^ The importance of the abundance of *Lactobacillus* in the endometrium is currently being debated. If lower *Lactobacillus* rates in the EM are associated with lower implantation rates, patients with repeated implantation failure would be expected to have abnormal incidence rates of *Lactobacillus* and high rates of pathogenic bacteria. In addition, we expected similar results for the vaginal microbiota (VM), because the vagina prevents the invasion of pathogens into the uterus. We compared the EM and VM communities between patients with RIF and healthy women at the genus level using next‐generation sequencing and analyzed the abundance of *Lactobacillus* and the presence of specific bacteria responsible for RIF.

## 2 MATERIALS AND METHODS

### 2.1 Patients

We diagnosed patients with RIF if they failed to achieve clinical pregnancy after at least three ET cycles, using the Gardner scoring system^15^ grade 3BB or higher blastocysts 5‐6 days post‐fertilization. A clinical pregnancy was considered when a fetal sac was observed in the uterus on transvaginal ultrasound. We enrolled 211 consecutive women diagnosed with RIF between October 2017 and June 2018. All patients' uterine and endometrial structures were evaluated via transvaginal ultrasonography. Sixty‐six patients with RIF were excluded for various reasons, including the presence of obvious risk factors for RIF. Patients with uterine cavity ultrasounds that revealed possible causes of infertility received a diagnostic hysteroscopy to rule out intrauterine disorders (eg, endometrial polyps, submucosal myomas, and intrauterine adhesions). Twenty‐nine women were excluded after ultrasound and hysteroscopy examinations. We also excluded 30 women with other possible risk factors for reproductive failure, including 13 with thrombophilia (eg, antiphospholipid syndrome), 15 with endocrinologic abnormalities or collagen disease, and 2 with parental chromosomal imbalances or translocations. Seven women who had received antibiotics within 1 month of sampling were excluded because antibiotics can affect microbiota communities.

**FIGURE 1:**
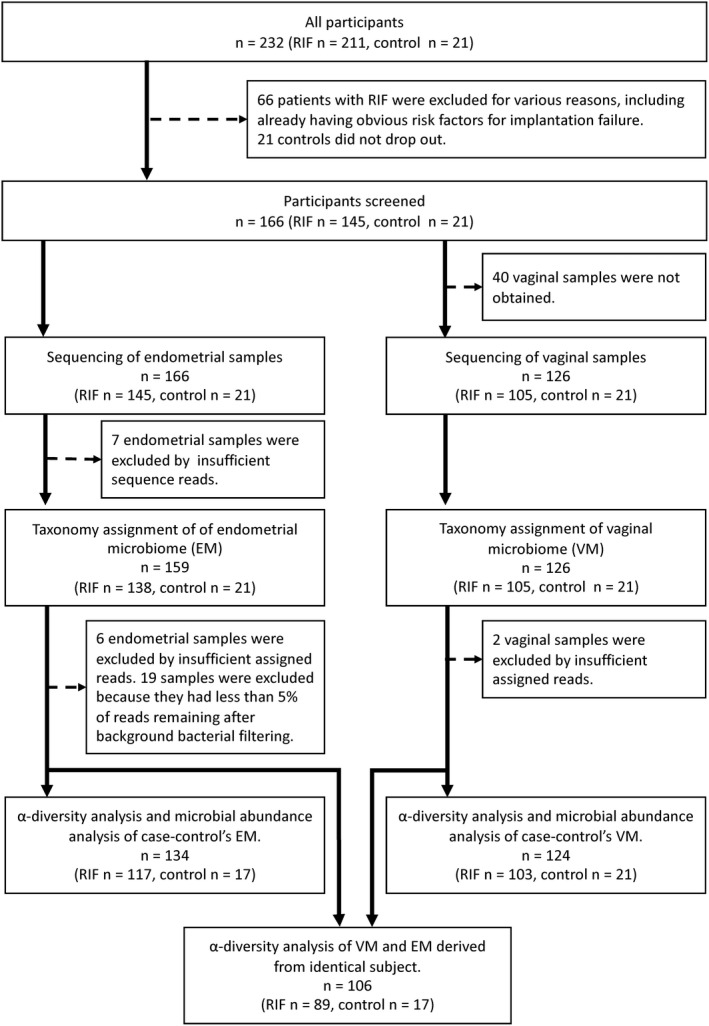
Study flowchart of the participants. Subsequent selection methods are shown

Finally, 145 women were included. Forty did not provide vaginal specimens; thus, we obtained 145 endometrial samples and 105 vaginal aspirates. We also recruited 21 healthy women with etiologies of infertility because their husbands had azoospermia as the control group; these women had regular menstrual cycles without causes of infertility such as tubal factors, ovulation disorder, endometriosis, endocrinologic abnormalities, or immunological abnormalities. Figure 1 shows the participant selection methods.

### 2.2 Vaginal and endometrial sampling

Both vaginal and endometrial samples were taken 5‐7 days after ovulation or the beginning of the high‐temperature phase in the basal body temperature. All the specimens were collected in a hormone‐free cycle, except in the four patients with irregular menstruation.

There were four women with irregular menstrual cycles in RIF group, and those samples were taken during the hormone replacement cycle. From days 1‐3 of the menstrual cycle, 2‐8 mg of oral estradiol valerate (Progynova^®^, Bayer Health Care, Schering, Germany) was administered. From day 13, oral chlormadinone acetate (8 mg; Lutoral, Shionogi, Osaka, Japan) was administered for 13 days. Samples were obtained 5‐7 days after initiating oral progesterone intake.

Vaginal discharge was first collected in the posterior fornix of the vagina using two sterilized swabs, after placing a sterilized vaginal speculum. One swab was submitted for Nugent scoring,^16^ which indicates BV; the other was used to analyze the microbiota. The latter swab was immediately soaked in OMNIgene^®^ VAGINAL (DNA Genotek Inc, Ottawa, Ontario, Canada). The vagina was then washed with physiological saline and wiped three times with dry tampons to remove vaginal secretions and cervical mucus. To minimize the risk of contaminating the endometrial samples in the vagina, a Medgyn pipette IV (Harada Corporation, Tokyo, Japan) was inserted into the uterine cavity, avoiding contact with the vaginal walls. If pipette insertion was difficult, we bent the pipette in the pack before insertion, following transabdominal ultrasonography to confirm the inclination of the uterus. We inserted the pipette approximately 5 mm from the bottom of the uterus and pulled it back toward the cervical canal under abdominal ultrasound guidance and absorbed the samples while turning the pipette slowly for 45‐60 seconds. All uterine samples were placed in an *in utero* solution with the endometrial tissue. We stopped the absorption to prevent uptake of any cervical mucus left after washing when the pipette neared the cervix, then quickly removed the pipette from the uterus. The pipette tip was cut to 3 cm with sterilized surgical scissors to prevent contamination with cervical mucus. The endometrial samples were placed into the OMNIgene^®^ VAGINAL without touching the pipette to the liquid. Participants for whom pipette insertion was difficult due to strong uterine flexion or other reasons were excluded from this study.

### 2.3 Nugent scoring

Nugent score was measured by Kyurin Corporation (Kitakyushu‐shi, Fukuoka, Japan). Vaginal samples were smeared on glass slides, fixed over a flame, and Gram stained. The stained slides were then examined at 1000× magnification to evaluate the Nugent scoring,^16^ an index for microscopically evaluating *Lactobacillus*, *Gardnerella* and *Mobiluncus* in vaginal samples. The scoring requires training but is a highly reproducible standard method, with scores ranging from 0: >30 lactobacilli or no *Gardnerella*‐like bacteria in the visual field to 4: no lactobacilli or >30 *Gardnerella*‐like bacterium in the visual field. *Mobiluncus* presence is an additional 2 points. Subjects with Nugent scores of 0‐3, 4‐6, and ≥7 points were categorized into the normal, intermediate, and BV groups, respectively.

### 2.4 DNA extraction and 16S rRNA sequencing

Varinos Inc, Shinagawa, Tokyo, Japan, extracted and sequenced the bacterial DNA. The vaginal and endometrial samples were treated with proteinase K and lysozyme solution per the manufacturer's instructions. Genomic DNA was extracted using an Agencourt Genfind v2 Blood & Serum DNA Isolation Kit (Beckman Coulter, Inc, Miami, FL, USA) or MagNA Pure 24 (Roche Diagnostics, Grenzach‐Wyhlen, Germany) Pathogen 200 hp 1.0 protocol. For DNA extraction, the test laboratory mainly carried out DNA extraction musing automated equipment, with manual DNA extraction if the specimens could not be properly processed by the automated equipment. Because two different DNA extraction methods were used, we confirmed that the two methods gave the same results for bacterial composition using previous clinical specimens. The dsDNA concentration was quantified fluorometrically with a Qubit dsDNA HS Assay Kit (Thermo Fisher Scientific Inc, Waltham, MA, USA). The V4 hypervariable region of the bacterial 16S rRNA gene was amplified from the specimen's DNA using the modified primer pair, 515f (5′‐TCG TCG GCA GCG TCA GAT GTG TAT AAG AGA CAG GTG YCA GCM GCC GCG GTA A‐3′) and 806rB (5′‐ GTC TCG TGG GCT CGG AGA TGT GTA TAA GAG ACA GGG ACT ACN VGG GTW TCT AAT‐3′), with Illumina Nextera XT adapter overhang sequences (underlined; Illumina, Inc, San Diego, CA, USA).^17^ PCR was performed using 25 ng/µL DNA, 200 µmol/L of each deoxynucleotide triphosphate, 400 nmol/L of each primer, 2.5 U FastStart HiFi polymerase, 4% of 20 mg/mL bovine serum albumin (Sigma‐Aldrich, St. Louis, MO, USA), 0.5 mol/L betaine (Sigma), and the appropriate buffer with MgCl_2_ supplied by the manufacturer (Roche Molecular Systems, Inc, Pleasanton, CA, USA). Thermal cycling consisted of initial denaturation at 94°C for 2 minutes, followed by 30 cycles of denaturation at 94°C for 20 seconds, annealing at 50°C for 30 seconds, extension at 72°C for 1 minute, and a final extension at 72°C for 5 minutes. The amplicon mixture was purified using Agencourt AMPure XP (Beckman Coulter, Inc). Purified PCR samples were multiplexed using a dual‐index approach with the Nextera XT Index kit v2 (Illumina, Inc) per the Illumina 16S Metagenomic Sequencing Library preparation protocol. PCR indexing was performed using KAPA HiFi HotStart ReadyMix (Roche Sequencing Solutions Inc, Pleasanton, CA, USA) in a 50‐µL reaction volume and subsequently purified using Agencourt AMPure XP beads. The final library was paired‐end sequenced at 2 × 200 bp using a MiSeq Reagent Kit v3 on the Illumina MiSeq platform. The ZymoBIOMICS Microbial Community Standard (Zymo Research, Irvine, CA, USA), containing a mixture of *Pseudomonas*, *Escherichia*, *Salmonella*, *Lactobacillus*, *Enterococcus*, *Listeria*, *Bacillus*, and two yeast species, was used as a positive control. UltraPure™ DNase/RNase‐Free Distilled Water (Thermo Fisher Scientific, Inc) was used as a blank control.

Sequenced reads were merged using EA‐Utils fastq‐join^18^ to obtain a 291‐bp median merged sequence length. Quality control of the merged sequence was performed using USEARCH v10.0.240^19^ to remove PhiX reads, truncate primer‐binding sequences, and discard sequences of <100 bp and with a sequence quality <Q20. Quantitative Insights Into Microbial Ecology (QIIME) 1.9.1 was used with the default parameters for quality filtering, chimera checking, sequence clustering into operational taxonomic units (OTUs), and taxonomic assignment.^20^ Sequences were clustered into OTUs using an open‐reference OTU‐picking strategy using the UCLUST method based on 97% sequence identity. Taxonomy was assigned to each OTU using RDP Classifier^21^ with a 0.50 confidence threshold against the Greengenes database, version 13_8.^22^ Taxonomy was determined at the genus level.

### 2.5 Sequencing results and operational taxonomic unit analysis

Seven endometrial samples with insufficient sequence reads were excluded. Thus, 39 716 693 reads were obtained from 159 endometrial samples and 126 vaginal samples. Seven endometrial samples with <1000 reads were excluded. The average read count per endometrial sample was 66 762 (range 192‐422 265) and per vaginal sample was 185 311 (range 203‐611 776). After quality filtering and OTU clustering, the average read counts were 11 742 (range 551‐42 236) and 38 368 (range 36‐46 155) for the endometrial and vaginal samples, respectively. Six endometrial samples and two vaginal samples with <1000 OTU hit reads were excluded. Low‐abundance taxa (0.01%) were filtered from the OTU tables (Figure 1). Bacterial taxa in a blank control were assumed to be contaminants from various reagents; therefore, blank‐characteristic taxa were subtracted to reduce background noise as in previous studies.^11^, ^23^ Fourteen bacterial taxa detected in a blank control and known to be reagent contaminants were excluded using QIIME: *Acidovorax*, *Acinetobacter*, *Chryseobacterium*, *Citrobacter*, *Escherichia*, *Flavobacterium*, *Janthinobacterium*, *Leptothrix*, *Methylobacterium*, *Pseudomonas*, *Rhodococcus*, *Sphingomonas*, *Stenotrophomonas*, and *Yersinia* (Table S1). Nineteen endometrial samples were excluded from the analysis because reads assigned to background bacteria accounted for >95% of all reads, and <5% of the reads remained after filtering.

### 2.6 Statistical analysis

We calculated the Shannon diversity index and Chao1 richness, which became the index of the microbiota's α‐diversity, then conducted *t* tests. We calculated the weighted UniFrac distance for analyzing the β‐diversity of the microbiotas between the samples and conducted PERMANOVA tests. The tests were analyzed using QIIME 1.9.1. We performed Welch's *t* tests using R 3.4.3 (https://www.r‐project.org/) to compare the bacterial abundances between groups. Hierarchical analysis was performed using R 3.4.3. Distances based on the squared Euclidean distance were calculated and clustered via Ward's method.

## 3 RESULTS

### 3.1 Study population

Table 1 shows the demographics of the control and RIF groups. The mean ages of the RIF and control groups were 38.3 ± 4.2 years and 32.0 ± 4.0 years, respectively (*P* < .001). Body mass index and smoking status showed no significant differences. RIF patients had more miscarriages than did the controls; thus, gravidity in the RIF group was higher than that of the controls, yet no significant difference occurred regarding parity. Nugent scores in the control and RIF groups were 0.9 ± 1.6 and 1.9 ± 2.7, respectively. No control patient was diagnosed with BV (Nugent score ≥7), whereas 11.9% of the RIF patients were diagnosed with BV. However, BV incidence and Nugent scores did not significantly differ (*P* = .13 and .09, respectively). Tables S2 and S3 compare the participants' characteristics for the vaginal and endometrial microbiome analyses, respectively.

**TABLE 1:** Participants' characteristics

	RIF n = 145	Control n = 21	*P*‐value
Age (years), mean ± SD	38.3 ± 4.2	32.0 ± 4.0	.68 × 10^−9^
Body mass index (kg/m^2^), mean ± SD	21.1 ± 2.8	21.2 ± 2.7	.92
Smoking, n (%)	2 (1.4)	1 (4.8)	.28
Pregnancy history, mean ± SD			
Gravidity	0.8 ± 1.3	0.1 ± 0.3	.01
Parity	0.1 ± 0.3	0	.14
Causes of infertility, n (%)			
Male factor	11 (7.6)	21 (100)	.22 × 10^−17^
Polycystic ovarian syndrome	5 (3.4)	0	.39
Endometriosis	19 (13.1)	0	.08
Tubal factor	13 (9.0)	0	.15
Unexplained infertility	100 (69.0)	0	.41 × 10^−11^
Previous history of ET, mean ± SD			
No of ET cycles	6.0 ± 4.6	0.3 ± 0.6	.46 × 10^−7^
No of transferred embryos	8.1 ± 7.2	0.4 ± 0.7	.29 × 10^−5^
No of ET cycles using morphologically good embryos	3.4 ± 2.1	0.1 ± 0.5	.34 × 10^−10^
No of transferred morphologically good embryos	4.0 ± 2.7	0.1 ± 0.5	.73 × 10^−9^
Nugent score, mean ± SD	1.9 ± 2.7	0.9 ± 1.6	.09
≥7 (Bacterial vaginosis), n (%)	16 (11.9)	0 (0)	.13

Note

Nugent score is often used for the diagnosis of bacterial vaginosis. Bacterial vaginosis is diagnosed as the score 7‐10.

Abbreviations: ET, embryo transfer; RIF, repeated implantation failure; SD, standard deviation.

### 3.2 Endometrial and vaginal microbiota bacterial diversities

**FIGURE 2:**
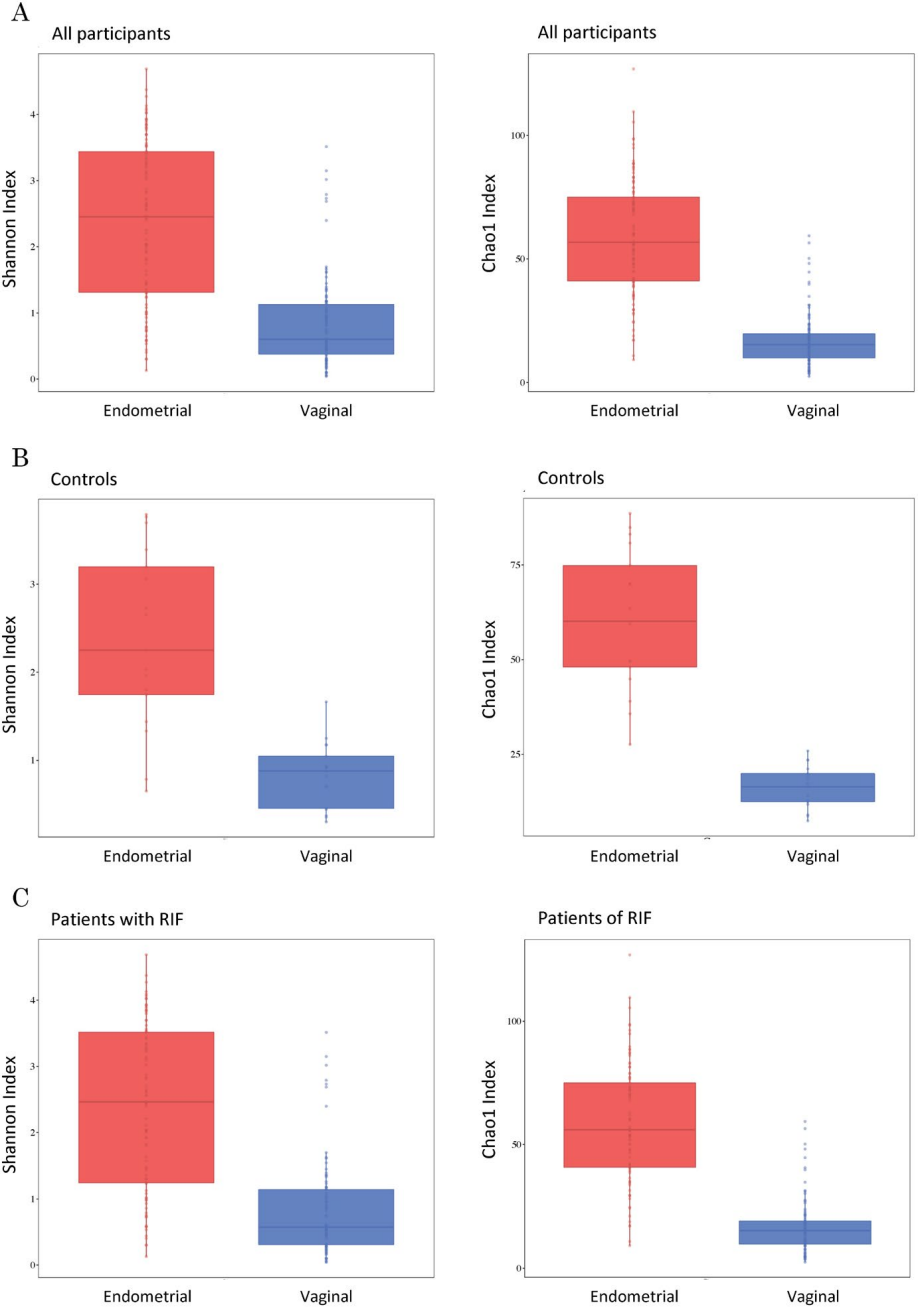
α‐diversities of the endometrial and vaginal bacterial compositions. Shannon diversity and Chao1 richness of the endometrial and vaginal microbiotas were calculated from the same individual from all participants (A), the controls (B) and RIF patients (C)

**FIGURE 3:**
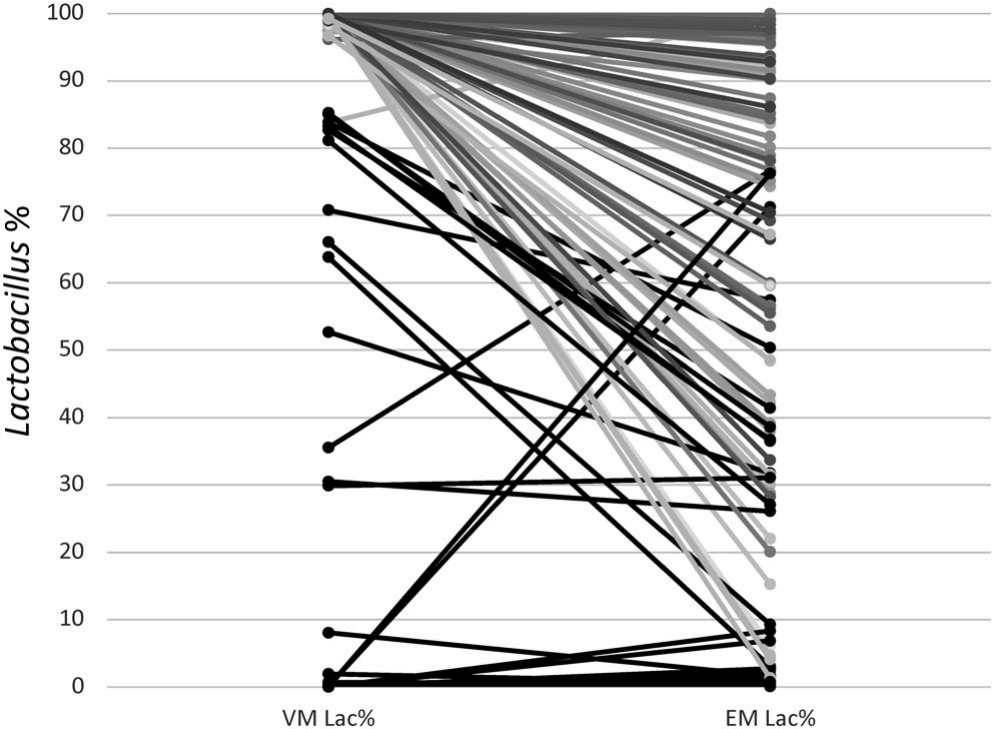
Comparison of *Lactobacillus* rates between vaginal and endometrial microbiotas from the same subject. From the 106 participants who provided both vaginal and endometrial samples, most (74 subjects) had a *Lactobacillus*‐rich vaginal microbiota (Lac% ≥90%). Thirty‐two of 74 subjects (43.2%) had vaginal and endometrial *Lactobacillus* rates that were both ≥90%. Conversely, 31/32 subjects (96.9%) had vaginal and endometrial *Lactobacillus* rates of ≤90%. Interestingly, when dysbiosis occurred in the vagina, it also occurred in the uterus

Shannon diversity and Chao1 richness indexes as α‐diversity metrics were calculated to compare the patients' vaginal and endometrial bacterial compositions (Table 2, Figure 2A). The Shannon diversity and Chao1 richness values in 1000 reads per sample were higher in the endometrial samples than in the vaginal samples (Shannon: 2.4 ± 1.2 and 0.8 ± 0.7, respectively, *P* < .001; Chao1: 59.1 ± 23.3 and 16.9 ± 10.9, respectively, *P* < .001). Regarding the vaginal and endometrial samples, both the control and RIF groups had higher EM diversities (Table 2, Figure 2B, Figure 1C). β‐diversity was analyzed to compare compositional dissimilarities between the EMs and VMs. Principal coordinate analysis (PCoA), the multivariate analysis based on weighted UniFrac distance to compare microbiome differences between groups, revealed significant associations between microbiotas (*P* = .001) (Figure S1). The few subjects had higher uterine *Lactobacillus* rates than vaginal *Lactobacillus* rates. Most subjects with lower vaginal *Lactobacillus* rates also had lower endometrial *Lactobacillus* rates; thus, individuals with vaginal dysbiosis also had uterine dysbiosis (Figure 3).

**TABLE 2:** Shannon index and Chao1 richness values of endometrial and vaginal microbiota derived from the identical subject

	Endometrial microbiota	Vaginal microbiota	*P*‐value
All women (n = 106), mean ± SD			
Shannon index	2.4 ± 1.2	0.8 ± 0.7	1.94 × 10^−22^
Chao1 richness	59.1 ± 23.3	16.9 ± 10.9	2.88 × 10^−36^
Control (n = 17), mean ± SD			
Shannon index	2.4 ± 1.0	0.8 ± 0.4	9.49 × 10^−06^
Chao1 richness	60.6 ± 18.5	16.1 ± 5.6	1.34 × 10^−08^
RIF (n = 89), mean ± SD			
Shannon index	2.4 ± 1.2	0.8 ± 0.7	4.29 × 10^−18^
Chao1 richness	58.8 ± 24.2	17.1 ± 11.7	5.61 × 10^−29^

Note

Shannon diversity and Chao1 richness values were calculated based on a subsample of 1000 sequences.

Abbreviations: RIF, repeated implantation failure; SD, standard deviation.

### 3.3 Bacterial community differences between the RIF and control groups

**FIGURE 4:**
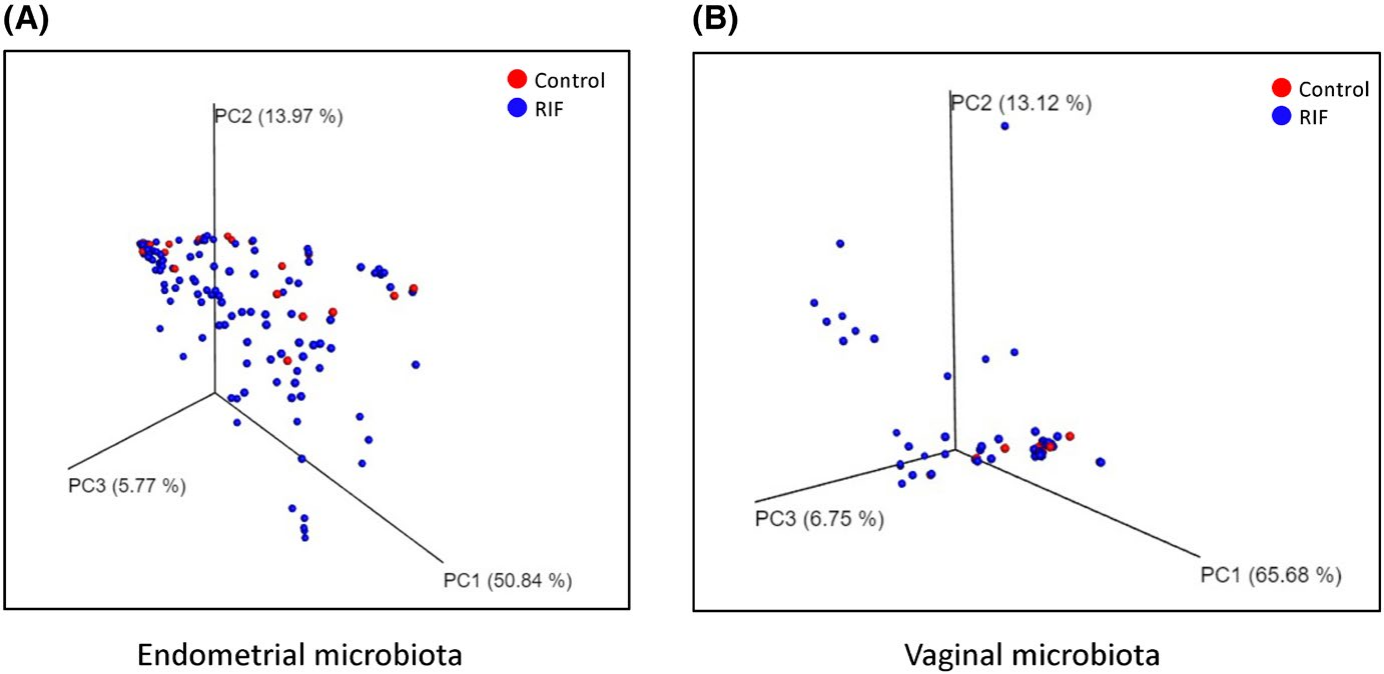
PCoA plot showing the relationship between the bacterial compositions of the controls and patients with RIF. (A) Principal coordinate analysis (PCoA) plot based on weighted UniFrac distance representing the endometrial microbiotas of the controls (red) and patients with repeated implantation failure (RIF) (blue). PCoA plot showing the relationship between the bacterial compositions of the endometrial and vaginal microbiotas. One red dot represents one control individual; one blue dot represents one RIF patient. A PERMANOVA test was conducted to compare the β‐diversity between the controls and RIF patients (*P* = .30). (B) PCoA plot based on the weighted UniFrac distance representing the vaginal microbiota (*P* = .053)

**FIGURE 5:**
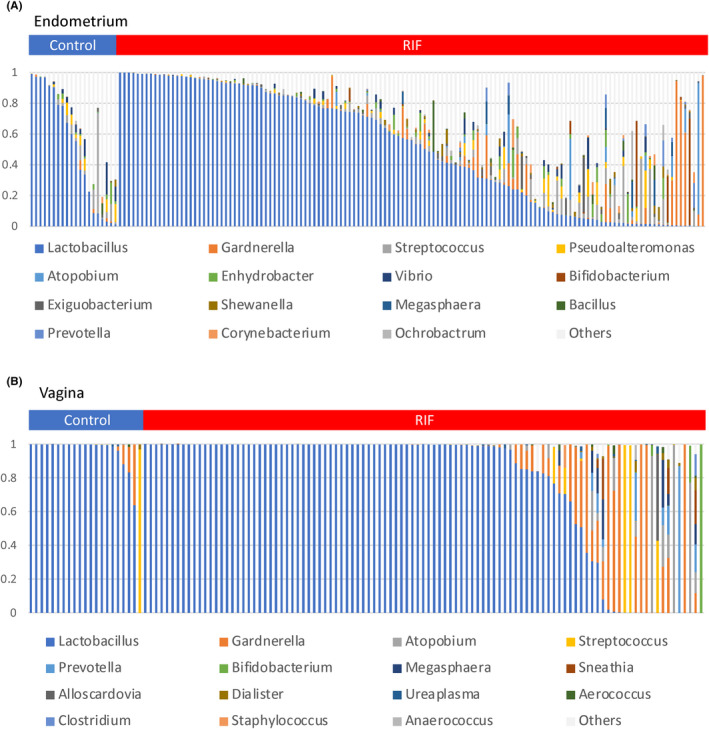
Bar charts of the bacterial species compositions in the vaginal and endometrial microbiotas of 166 participants. Endometrial (A) and vaginal (B) microbiotas. Twenty‐five bacterial species were detected from the vagina, and 131 were detected from the uterus. The top 15 bacterial species are displayed. One bar on the horizontal axis represents one sample. The vertical axis represents the bacterial abundance in the microbiota

To identify the relationship between bacterial diversity and implantation failure, we compared the microbiota data from the endometrial and vaginal samples between the RIF and control groups (Table S4). The α‐ and β‐diversities did not significantly differ in the EMs or VMs between the RIF and control groups (Figure 4A,B). We further investigated differences between the bacterial genera in these groups. Twenty‐five and 131 bacterial species were detected from the vaginal and endometrial samples, respectively. Figure 5 shows the relative bacterial abundances in the top 15 bacterial species with the highest proportions. *Lactobacillus* dominated in both groups and sample types. The uneven height of the bar chart for the endometrium indicates that many bacterial species other than the top 15 were detected.

To identify candidate bacterial genera as risk factors for RIF, bacterial abundances in the EMs and VMs were evaluated (Table 3). For bacterial species with average abundances of >1.0%, the VMs in the RIF patients had higher rates of *Atopobium*, *Megasphaera*, *Gardnerella*, and *Prevotella* than did the control group. The *Lactobacillus* rate in the RIF group VMs was significantly lower than that in the controls (76.4 ± 77.7% and 91.8 ± 45.5%, respectively, *P* = .015). Of the EMs in the RIF group, 14 genera (*Atopobium, Megasphaera, Gardnerella, Prevotella, Schlegelella, Delftia, Burkholderia*, *Sphingobacterium*, *Dietzia*, *Enterococcus*, *Micrococcus*, *Ralstonia*, *Leucobacter*, and *Hydrogenophaga*) were significantly higher than those in the controls (Table 3). The endometrial *Lactobacillus* abundances did not significantly differ between the RIF and control groups (51.2 ± 37.5% and 51.6 ± 38.3%, respectively).

**TABLE 3:** Comparison of microbial genera between RIF and control groups

Taxonomy, % mean ± SD	Endometrial microbiota			Vaginal microbiota		
	RIF n = 117	Control n = 17	*P*‐value	RIF n = 103	Control n = 21	*P*‐value
*Atopobium*	2.1 ± 9.4	0.1 ± 0.2	.**025**	3.9 ± 15.7	0 ± 0	.**014**
*Megasphaera*	0.8 ± 3.2	0 ± 0	.**009**	1.0 ± 4.3	0 ± 0	.**016**
*Lactobacillus*	56.2 ± 36.4	58.8 ± 37	.794	76.4 ± 38.9	91.8 ± 22.7	.**018**
*Gardnerella*	5.3 ± 16.3	0.6 ± 1.6	.**003**	10 ± 24.2	3.1 ± 8.6	.**025**
*Prevotella*	0.7 ± 2.6	0 ± 0.1	.**009**	1.9 ± 9.4	0 ± 0.1	.**048**
*Schlegelella*	0.4 ± 1.1	0 ± 0	.**001**	‐	‐	**‐**
*Delftia*	0.2 ± 0.3	0 ± 0.1	.**001**	0 ± 0.2	0 ± 0	.316
*Burkholderia*	0.5 ± 1.3	0.1 ± 0.2	.**003**	‐	‐	‐
*Sphingobacterium*	0.3 ± 1.1	0 ± 0	.**005**	‐	‐	‐
*Dietzia*	0.1 ± 0.5	0 ± 0	.**017**	‐	‐	‐
*Enterococcus*	0.1 ± 0.3	0 ± 0	.**025**	0.1 ± 0.8	0 ± 0	.316
*Micrococcus*	0.1 ± 0.7	0 ± 0	.**033**	‐	‐	‐
*Ralstonia*	0.3 ± 1.2	0 ± 0.1	.**034**	‐	‐	‐
*Leucobacter*	0.2 ± 0.6	0.1 ± 0.2	.**035**	‐	‐	‐
*Hydrogenophaga*	0.1 ± 0.3	0 ± 0	.**043**	‐	‐	‐

Note

Bold text indicates a statistically significant difference with a *P*‐value less than .05.

Abbreviations: RIF, repeated implantation failure; SD, standard deviation.

## 4 DISCUSSION

Although the uterus was once hypothesized to be sterile via the cervical mucus,^24^, ^25^ intrauterine bacterial microbiotas have since been confirmed.^12^, ^26^, ^27^ In the endometrial cells of fertile women, progesterone secretion from the luteum body induces subnuclear vacuole production, leading to increased glycogen levels.^28^ Deposition of endometrial epithelial glycogen may allow bacteria to colonize the endometrium.^28^ We found that the EMs had higher α‐diversity than did the VMs, as previously reported.^29^ Lactic acid produced by *Lactobacillus* acidifies the vagina, thus inhibiting the growth of other bacterial species^30^, ^31^; however, the number of bacteria in the uterus is extremely small at 1/100‐1/10 000 that of the vagina, and some bacterial species dominate among the highly varied vaginal bacteria, leading to low bacterial diversity in the vagina.^32^, ^33^ Therefore, the EM community is mostly independent of the VM community.

A healthy microbiota generated by a healthy lifestyle is defined as “eubiosis,” and disruption of this balance inclines toward a state of “dysbiosis,” in which pathogenic bacteria predominate over endogenous bacteria due to an inappropriate immune response, inflammation, or suppressed immune response.^34^ The cervical mucus plug is partially impermeable to bacterial ascension from the vagina^35^, ^36^; therefore, some vaginal bacteria can translocate to the endometrial microenvironment. Assuming that the genital microbiota in a healthy, young control represented eubiosis, our results demonstrated that RIF was associated with high incidences of dysbiosis, which is a microbiota community imbalance, in the both VMs and EMs compared with that of the controls. In the vagina, high *Atopobium*, *Megasphaera*, *Gardnerella*, and *Prevotella* and low *Lactobacillus* levels were associated with RIF. Among these genera, *Atopobium*, *Gardnerella, Prevotella,* and *Megasphaera* have been reported as pathogenic bacteria involved in BV.^37^, ^38^, ^39^, ^40^ Furthermore, the reduced vaginal *Lactobacillus* rate can trigger pathological bacterial overgrowth in BV.^37^ Therefore, vaginal dysbiosis, including BV with low *Lactobacillus* rates, may be a biomarker for implantation failure. Fu et al^41^ also reported the relationship between RIF and vaginal microbial dysbiosis.

For the EMs, 14 bacterial genera were detected as possible risk factors for RIF. Among them, *Atopobium*, *Gardnerella, Prevotella,* and *Megasphaera* were the same as the candidate vaginal bacteria as biomarkers for RIF; therefore, they might ascend from the vagina. Although nine other species, excluding *Burkholderia*, were significantly detected from the endometrial samples of RIF patients, the occupancy rate of these bacteria was <0.5%. The clinical role that these bacteria play in reproductive‐aged women (excluding compromised hosts) remains unknown and requires further analysis. Kitaya et al^29^ detected significant vaginal *Burkholderia* levels in RIF patients; however, endometrial, but not vaginal, *Burkholderia* abundances differed significantly between RIF patients and controls in our study. Recently, researchers detected *Burkholderia* in a preterm delivery and a tubo‐ovarian abscess^42^, ^43^; thus, *Burkholderia* in female reproductive organs may be associated with RIF and thus a treatment target.

Interestingly, endometrial *Lactobacillus* abundances did not significantly differ between RIF patients and controls. Moreno et al^12^ reported that high *Lactobacillus* abundances (≥90%) in EMs were associated with good pregnancy prognoses after IVF. However, in our study, only six women (28.6%) in the control group had ≥90% *Lactobacillus* abundances in the EM. Therefore, ≥90% *Lactobacillus* abundance is not a biomarker for implantation failure. Kyono et al^14^ reported that pregnancy outcomes after IVF in infertile patients did not significantly differ between those with and without ≥90% *Lactobacillus* in the EMs. A normal range for endometrial *Lactobacillus* rates should be reanalyzed in fertile women when reconsidering the endometrial *Lactobacillus* abundance as a biomarker for RIF.

Dysbiosis or BV with pathogenic bacteria (eg, *Atopobium, Megasphaera, Gardnerella,* and *Prevotella*) in the VMs may suggest endometrial dysbiosis of vaginally derived bacteria, leading to implantation failure; thus, these bacteria may be candidate biomarkers for RIF. Sampling endometrial specimens is often invasive and carries a risk of intrauterine infection, whereas collecting vaginal samples is easy and reproducible; thus, VMs yield more stable results. Some pathogenic bacteria, such as *Megasphaera*, are difficult to detect on common bacterial cultures^39^, ^40^; thus, microbiome analysis may be indispensable for patients with RIF. BV is associated with obstetric complications such as preterm birth and midterm abortion^5^, ^6^, ^7^, ^8^, ^9^, ^10^, ^44^; therefore, treating vaginal dysbiosis may help improve embryo receptivity and prevent complications post‐pregnancy.

This study had some limitations. First, we selected healthy women as the super control group, who do not possess the factors responsible for RIF and have high probability of pregnancy, but no experience of childbirth due to their partners' diagnosis of azoospermia. Endometrial sampling is invasive; as a volunteer, the sample should not be collected from women capable of pregnancy, especially from those with birth history. The sample size of the control group was thus inevitably small. Second, the women in the control group were significantly younger than the infertile women who had undergone IVF in the RIF group. Although both the controls and RIF patients were of reproductive age, bias may have occurred between the RIF and control groups owing to age‐related changes in the vaginal and intrauterine environments. Third, the samples of four patients with PCOS were collected at the time of the luteal replacement starts, while using the hormone (drugs) as same as the basic procedure. There is a report that shows to control the menstrual cycle using the hormone has some effects on the microbiota. Therefore, the research may have been influenced by the presence of the hormone.^45^ Fourth, it was difficult to prove that a sterile organ, such as the endometrium, was completely free of contamination. The amount and nature of the cervical mucus change with the phase of menstruation, and the amount of cervical mucus increases and is highly glutinous in the growth phase, making it difficult to completely remove the samples, even if they are washed or wiped with a tampon. We therefore collected the samples in the secretory phases, being careful to prevent contamination. If we detected cervical mucus on the tip of the pipette immediately after collecting the endometrial sample, even after careful washing, we cut off the tip of the pipette; however, the usefulness of this procedure has not been proven.

The vaginal and endometrial environments had individual microbiota profiles. However, RIF presented high incidences of dysbiosis with *Atopobium*, *Gardnerella*, *Prevotella,* and *Megasphaera* in both the VMs and EMs; therefore, vaginal dysbiosis including BV may affect endometrial microbiota communities, leading to implantation failure. In addition, vaginal, but not endometrial, *Lactobacillus* abundances were associated with RIF. Therefore, treating vaginal pathogenic bacteria may improve endometrial receptivity in infertile patients with histories of RIF. The vaginal microbial profiles of patients with endometrial and vaginal dysbiosis require further analysis, and treatments are needed.

## DISCLOSURES

*Conflict of interest*: The authors declare that they have no conflict of interest. *Human rights statement and informed consent*: The Ethical Committee of the Institutional Review Board of Saint Mother Obstetrics and Gynecology Clinic and Institute for ART, Fukuoka, Japan, approved this study in November 2017 (No. 17‐ST‐03). The study was registered in the University Hospital Medical Information Network‐Clinical Trial Registration, Japan, in March 2018 (UMIN‐CTR000031731). All procedures followed were in accordance with the ethical standards of the responsible committee on human experimentation (institutional and national) and with the Helsinki Declaration of 1964 and its later amendments. All recruited women provided written informed consent. The data that support the findings of this study are available on request from the corresponding author. The data are not publicly available due to privacy or ethical restrictions. *Animal studies*: This article does not contain any study with animal participants that have been performed by any of the authors.

## Supporting information

Fig S1

Table S1

Fig S1‐caption
