# Supplementary figures and images for: Analysis of vaginal and endometrial microbiota communities in infertile women with a history of repeated implantation failure
Source: Reprod Med Biol. 2021 May 31;20(3):334–44. doi: 10.1002/rmb2.12389 (PMC8254176; doi:10.1002/rmb2.12389)

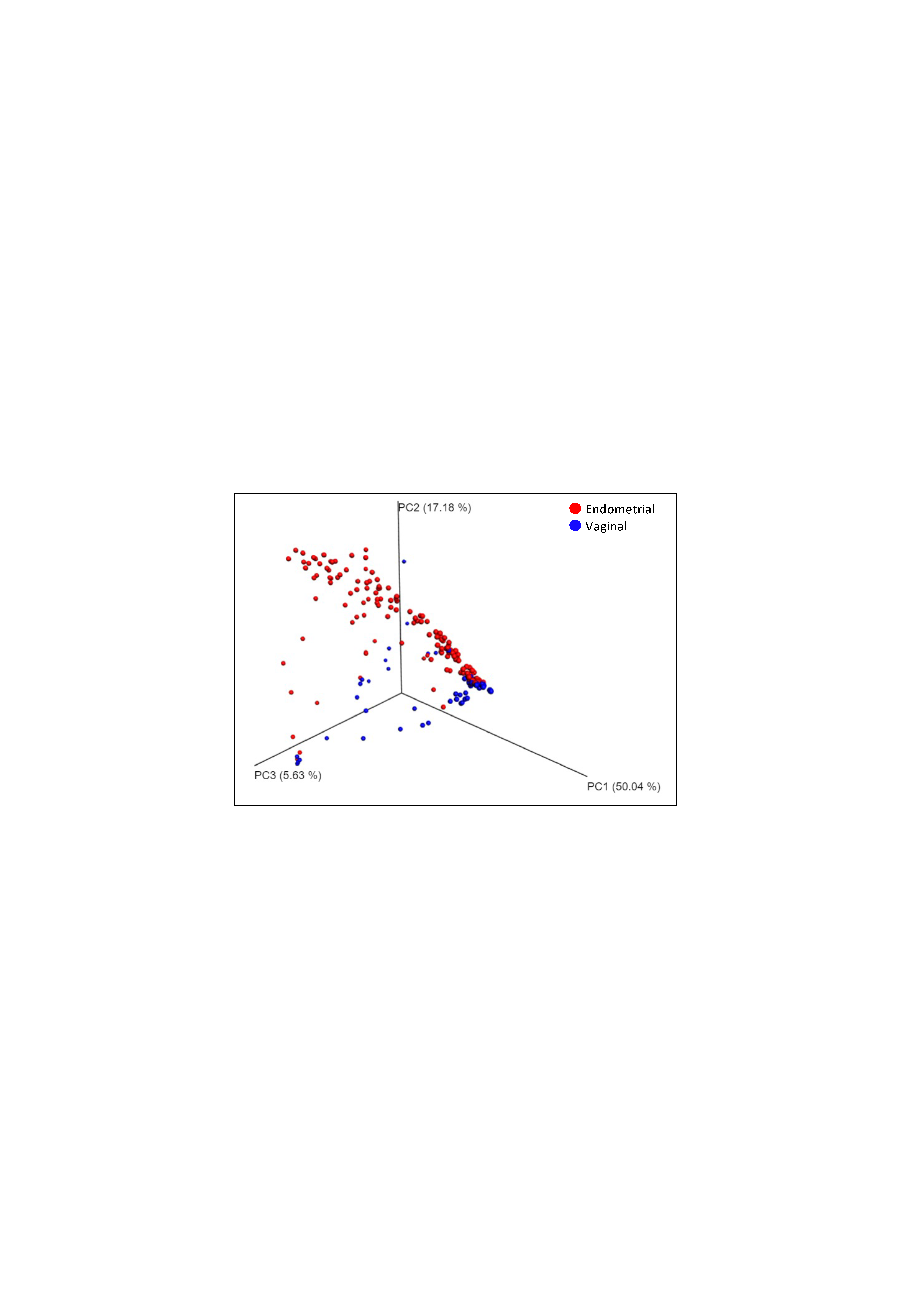

Supplement: Supplementary file 1 — Fig S1 [file RMB2-20-334-s002.tiff]
