# Supplementary material for: Analysis of vaginal and endometrial microbiota communities in infertile women with a history of repeated implantation failure
Source: Reprod Med Biol. 2021 May 31;20(3):334–44. doi: 10.1002/rmb2.12389 (PMC8254176; doi:10.1002/rmb2.12389)
Supplement: Supplementary file 2 — Table S1 [file RMB2-20-334-s001.docx]

Supplemental Table 1. Microbial genera detected in blank controls.

|  | **Manual protocol batch 1 (n = 10)** | | **Manual protocol batch 2 (n = 14)** | |
| --- | --- | --- | --- | --- |
| **Taxonomy** | **Mean abundance (%)** | **Detection**  **rate (%)** | **Mean abundance (%)** | **Detection**  **rate (%)** |
| ***Pseudomonas*** | 20.7 | 10/10 (100) | 18.9 | 14/14 (100) |
| ***Escherichia*** | 41.3 | 10/10 (100) | 17.6 | 14/14 (100) |
| ***Rhodococcus*** | 2.5 | 8/10 (80) | 0.1 | 1/14 (7.1) |
| ***Janthinobacterium*** | 2.8 | 10/10 (100) | 7.4 | 13/14 (92.9) |
| ***Sphingomonas*** | 4.4 | 10/10 (100) | 0.6 | 7/14 (50) |
| ***Flavobacterium*** | 3.6 | 9/10 (90) | 0.8 | 12/14 (85.7) |
| ***Methylobacterium*** | 1.8 | 1/10 (10) | 0.4 | 2/14 (14.3) |
| ***Stenotrophomonas*** | 2.5 | 8/10 (80) | 0.6 | 9/14 (64.3) |
| ***Acinetobacter*** | 1.9 | 3/10 (30) | 0.6 | 10/14 (71.4) |
| ***Leptothrix*** | 0.0 | 0/10 (0) | 1.1 | 9/14 (64.3) |
| ***Acidovorax*** | 0.0 | 0/10 (0) | 0.1 | 3/14 (21.4) |
| ***Chryseobacterium*** | 0.0 | 0 /10 (0) | 1.1 | 12/14 (85.7) |
| ***Citrobacter*** | 0.0 | 0 /10 (0) | 0.4 | 9/14 (64.3) |
| ***Yersinia*** | 0.0 | 0 /10 (0) | 48.0 | 14/14 (100) |

The composition of the bacteria detected from the blank controls varied with each lot of reagents, and bacteria detected from multiple lots were used as background bacteria.

Supplemental Table 2. Characteristics of participants for vaginal microbiome analysis

|  | RIF  n = 103 | Control  n = 21 | *p*-value |
| --- | --- | --- | --- |
| **Age (years), mean ± SD** | 38.1 ± 4.1 | 32.0 ± 4.0 | 0.50x10^-8^ |
| **Body mass index (kg/m2), mean ± SD** | 20.9 ± 2.6 | 21.2 ± 2.7 | 0.65 |
| **Smoking, n (%)** | 2 (1.9) | 1 (4.8) | 0.43 |
| **Pregnancy history, mean ± SD**  Gravidity  Parity | 0.7 ± 1.2  0.2 ± 0.4 | 0.1 ± 0.3  0 | 0.02  0.08 |
| **Causes of infertility, n (%)**  Male factor  Polycystic ovarian syndrome  Endometriosis  Tubal factor  Unexplained infertility | 9 (8.7)  3 (2.9)  13 (12.6)  10 (9.7)  69(67.0) | 21 (100)  0  0  0  0 | 0.22x10^-15^  1  0.12  0.21  0.28x10^-8^ |
| **Previous history of ET, mean ± SD**  No of ET cycles  No of transferred embryos  No of ET cycles using morphologically good embryos  No of transferred morphologically good embryos | 5.6 ± 4.1  7.5 ± 6.7  3.3 ± 2.0  3.9 ± 2.3 | 0.3 ± 0.6  0.4 ± 0.7  0.1 ± 0.5  0.1 ± 0.5 | 0.37ｘ10^-7^  0.42ｘ10^-5^  0.46x10^-10^  0.10x10^-10^ |
| **Nugent score, mean ± SD**  **≥7 (Bacterial vaginosis), n (%)** | 2.1 ± 2.8  14 (13.6) | 0.9 ±1.6  0 (0) | 0.05  0.12 |

RIF = repeated implantation failure

ET = embryo transfer

SD = standard deviation

Nugent score is often used for the diagnosis of bacterial vaginosis. Bacterial vaginosis is diagnosed as the score 7-10.

Supplemental Table 3. Characteristics of participants for endometrial microbiome analysis

|  | RIF  n = 117 | Control  n = 17 | *p*-value |
| --- | --- | --- | --- |
| **Age (years), mean ± SD** | 38.0 ± 4.2 | 32.5 ± 4.2 | 0.90x10^-6^ |
| **Body mass index (kg/m2), mean ± SD** | 21.3 ± 2.9 | 21.7 ± 2.7 | 0.56 |
| **Smoking, n (%)** | 2 (1.7) | 0 (0) | 1 |
| **Pregnancy history, mean ± SD**  Gravidity  Parity | 0.7 ± 1.2  0.2 ± 0.4 | 0.1 ± 0.2  0 | 0.02  0.10 |
| **Causes of infertility, n (%)**  Male factor  Polycystic ovarian syndrome  Endometriosis  Tubal factor  Unexplained infertility | 10 (8.5)  4 (3.4)  15 (12.8)  10 (8.5)  78 (66.7) | 17 (100)  0  0  0  0 | 0.60x10^-14^  1  0.22  0.36  0.69x10^-7^ |
| **Previous history of ET, mean ± SD**  No of ET cycles  No of transferred embryos  No of ET cycles using morphologically good embryos  No of transferred morphologically good embryos | 5.8 ± 4.1  7.8 ± 6.8  3.4 ± 2.1  3.9 ± 2.6 | 0.3 ± 0.6  0.4 ± 0.8  0.1 ± 0.5  0.1 ± 0.5 | 0.18ｘ10^-6^  0.16ｘ10^-4^  0.24x10^-8^  0.26x10^-7^ |
| **Nugent score, mean ± SD**  **≥7 (Bacterial vaginosis), n (%)** | 2.1 ± 2.9  15 (12.9) | 1.0 ±1.7  0 (0) | 0.14  0.22 |

RIF = repeated implantation failure

ET = embryo transfer

SD = standard deviation

Nugent score is often used for the diagnosis of bacterial vaginosis. Bacterial vaginosis is diagnosed as the score 7-10.

Supplemental Table 4. Comparison of α-diversity between control and RIF groups

|  | RIF | Control | *p*-value |
| --- | --- | --- | --- |
| **α-diversity of endometrial microbiota** | n = 117 | n = 17 |  |
| Shannon diversity, mean ± SD | 2.4 ± 1.2 | 2.4 ± 1.0 | 0.99 |
| Chao1 richness, mean ± SD | 59.1 ± 22.8 | 60.6 ± 18.0 | 0.8 |
| **α-diversity of vaginal microbiota** | n = 103 | n = 21 |  |
| Shannon diversity, mean ± SD | 0.9 ± 0.7 | 0.9 ± 0.4 | 0.99 |
| Chao1 richness, mean ± SD | 17.7 ± 12.0 | 16.8 ± 6.7 | 0.63 |

RIF = repeated implantation failure

SD = standard deviation
