# Supplementary material for: Analysis of vaginal and endometrial microbiota communities in infertile women with a history of repeated implantation failure
Source: Reprod Med Biol. 2021 May 31;20(3):334–44. doi: 10.1002/rmb2.12389 (PMC8254176; doi:10.1002/rmb2.12389)
Supplement: Supplementary file 3 — Fig S1‐caption [file RMB2-20-334-s003.docx]

Supplemental Figure 1. PCoA plot showing the relationship between the bacterial compositions of the vagina and endometrium
